# Supplementary material for: Exposure to Nanoplastic Particles Enhances Acinetobacter Survival, Biofilm Formation, and Serum Resistance
Source: Nanomaterials (Basel). 2022 Nov 27;12(23):4222. doi: 10.3390/nano12234222 (PMC9735686; doi:10.3390/nano12234222)
Supplement: Supplementary file 1 [file nanomaterials-12-04222-s001.zip › nanomaterials-2011570-supplementary.pdf]

Supplementary Materials

Exposure to Nanoplastic Particles Enhances *Acinetobacter* Survival, Biofilm Formation, and Serum Resistance

Mingfeng Tang [1], Guoying Ding [1], Xiaoyu Lu [1], Qian Huang [1], Huihui Du [1], Guosheng Xiao [1,*] and Dayong Wang [2,*]

[1] College of Biology and Food Engineering, Chongqing Three Gorges University, Wanzhou 404100, China
[2] Medical School, Southeast University, Nanjing 210009, China
* Correspondence: xgs03@sanxiau.edu.cn (G.X.); dayongw@seu.edu.cn (D.W.)

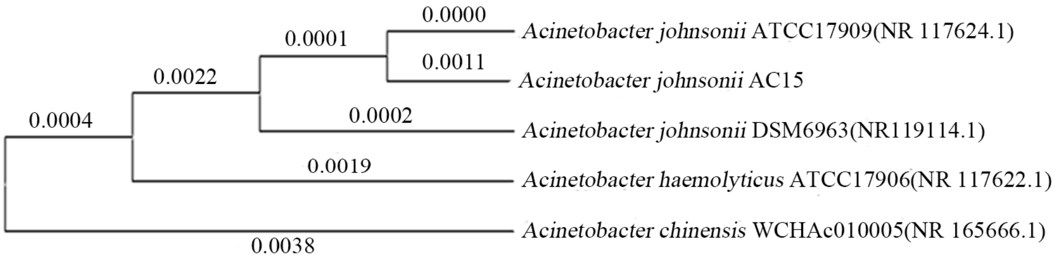

Figure S1. Systematic evolutionary tree including AC15. The form of labeled tree was used. After the sequencing, the data was compared with the published sequences in GeneBank. Using BLAST in NCBI website, the systematic evolutionary tree was constructed. In this evolutionary tree, AC15 was identified as *Acinetobacter Johnson*.

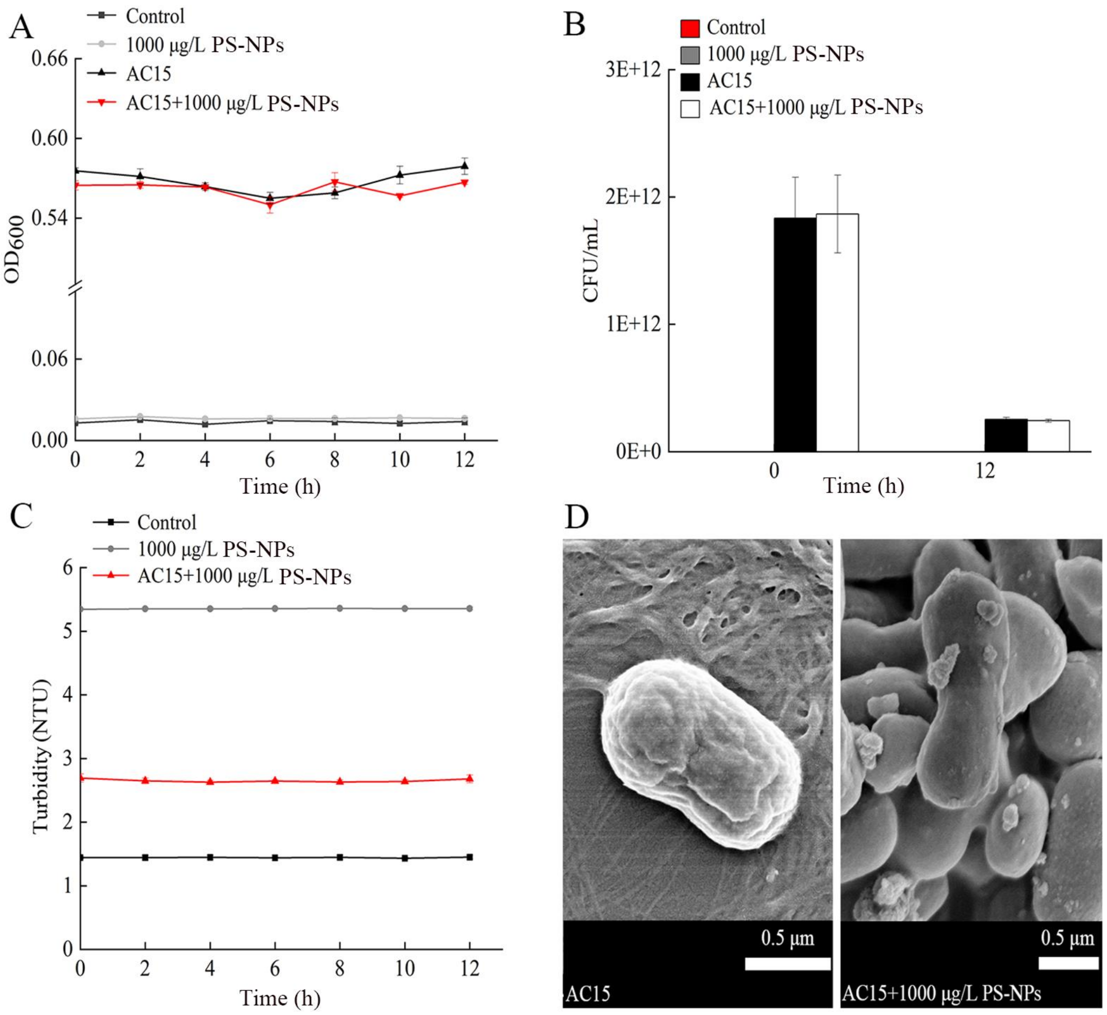

Figure S2. Adsorption curve of NPs under the condition of bacterial non-growth. (A) AC15 growth curve after exposure to 1000 μg/L NP. (B) Effect of 1000 μg/L NP exposure on AC15 CFU at different times. (C) Turbidity analysis of bacterial solutions after cultivation at 4 °C for 12 h. (D) SEM analysis to show NPs accumulation on surface of AC15 under the condition of 4 °C cultivation.

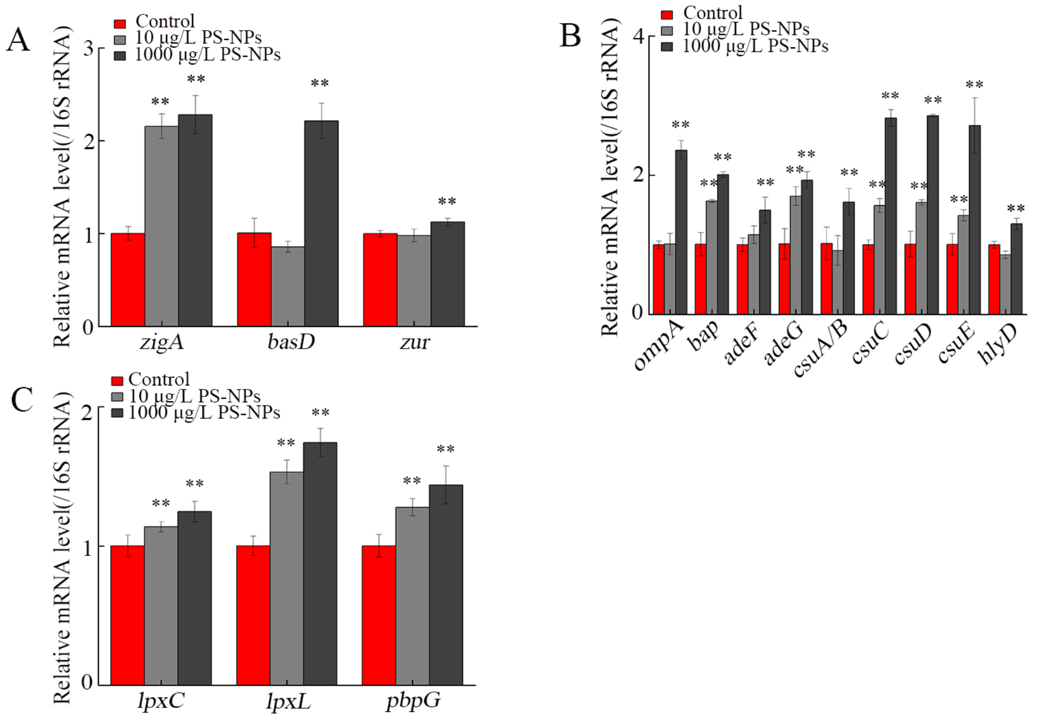

Figure S3. Effect of exposure to NPs on expression of virulence genes in AC15 in NHS. (A) Survival related genes. (B) Biofilm formation related genes. (C) Serum resistance related genes. $^{**}P < 0.01$ *vs* Control.

Table S1. Primer information for qRT-PCR

Gene	Forward primer (5'-3')	Reverse primer (5'-3')
16srRNA	CCTACCAAGGCGACGATCTGT	AACGCTCGCACCCTCTGTATT
zigA	GTGAGCATACGCCAGAGACTGAAG	TTTAAGCCTTAGGACGCACGACTC
basD	ACATGGGAACCTGAACGTGCTTC	ATACGGTGATATTCCAAGGCGACTG
zur	CAAGCAGCATTCCTGATTTGTACCG	TGTTGAGCCACGAAGTCATCTGAAG
ompA	TCAGCTTCGAAACCTAACCATGG	CAAGCAGCATTCCTGATTTGTACCG
bap	AACTGCGGATATGGCGAATGTAGG	AGTGACATCAACATCTGGCGAACC
adeF	AATAGCGGATTACAAGCGGGTGATC	CGGAACGAGATGGGTGTAACAGG
adeG	GGTTCTGGTGACTACGCGATGC	TTGTACCCGCTGCAACCTGAATG
csuA/B	TAACGTTTACCGCGATGCTGCTC	TTCAATTGGTACGGAGTAGCTTGGC
csuC	AGTTACCGATTCGCCTTTCTGATGG	CACTGCCAATTCCTTTCCCATAAGC
csuD	TGACGCGACGCTTTATGAGATAGTG	AAGCGAACCAGATAGACCAAACCAG
csuE	TGGGAATCCGTTGCTCGGTAAATAC	TGACTTCATGGCTCGCTGGTTG
hlyD	CTGCTGCGGGTTCTGAGTTCAG	GCCAGGGCGTAGTGTTCTATTCC
lpxC	TTCCGCATTGCCTGTGAGATGG	ATCTCCCGTTACCAAGTTGGAACAC
lpxL	GCCCGATCAGACTTTGGACTCAAG	AACGACGATGAGCAGTTACTGTGG
pbpG	CTCGTGCCTTGGGTATGAGTTCG	ATGGTAAGTCGGTACAACGTGATGG
